# Jaundice caused by protrusion of a hepatic cyst into common bile duct that was resolved by choledochoscopic needle-knife electrotomy: a case report

**DOI:** 10.1186/s12876-018-0815-x

**Published:** 2018-06-19

**Authors:** Cheng Zhang, Yue-Feng Ma, Yu-Long Yang

**Affiliations:** 1 Department of Biliary Minimally Invasive Surgery, Affiliated Zhongshan Hospital of Dalian University, Dalian, 116001 Liaoning Province China; 2 Cholelithiasis Center, East Hospital of Tongji University, 150 Jimo Road, Pudong, Shanghai, 200120 China

**Keywords:** Hepatic cyst, Obstructive jaundice, Choledochoscope, Electrotomy

## Abstract

**Backgroud:**

Hepatic cysts are the most frequent, innocuous, space-occupying lesions of the liver. The majority of solitary liver cysts are nonsymptomatic. When liver cysts reach a large size, there are some complications, including infection, rupture, spontaneous hemorrhage, obstructive jaundice, and neoplastic degeneration. Percutaneous aspiration, fenestration, hepatic resection, and liver transplantation have been proposed for symptomatic patients.

**Case presentation:**

In this case report, we describe a 41-year-old woman who presented with persistent liver dysfunction, indolent xanthochromia, and skin itching for 3 months. After a series of tests, she has a 5.0 × 5.3 cm hepatic cyst with many separations in the left medial liver lobe. The obstructive jaundice was caused by a large pedunculated lump protruding into the common bile duct from the left hepatic duct. She was treated with laparotomy and this lump was completely removed from the root by choledochoscopic needle-knife electrotomy with a good clinical response. Postoperative pathology of the lump suggested a hepatic cyst wall without heterocysts or tumor cells.

**Conclusion:**

Hepatic cyst wall protruding into the common bile duct can form capsular lump and result in indolent jaundice. Choledochoscopic high-frequency needle-knife electrotomy could be considered as a simple, safe and effective complementary approach for benign mass on the bile duct wall.

## Backgroud

Hepatic cysts consist of a heterogeneous group of disorders, which can be classified by etiology into congenital, traumatic, infectious, or neoplastic cysts. Congenital hepatic cysts are a result of congenital anomalies of the biliary system, including simple cysts and adult polycystic liver disease in which the liver is diffusely occupied by cysts. Generally, simple hepatic cysts contain fluid, and their inner walls are covered with a layer of epithelial cells.

Based on the anatomical features and modern imaging equipment, the clinical diagnosis of simple liver cysts is relatively easy and accurate. Ultrasonography (US) shows a well-circumscribed anechoic lesion with increased through-transmission of sound and no evidence of mural nodularity. Computed tomography (CT) shows water-density lesions with sharply defined margins and smooth thin walls, and Magnetic resonance imaging (MRI) shows homogeneously hypointense lesions on T1-weighted imaging and these are homogeneously hyperintense on T2-weighted imaging [1].

Simple cysts are usually frequent and asymptomatic, but when cysts are larger than 10 cm in diameter, pressure symptoms may occur in the surrounding organs [1]. Many studies have reported pressure symptoms caused by hepatic cysts, including portal hypertension, edema due to caval compression, jaundice, arrhythmia, and duodenal obstruction, which are accompanied by infection spontaneous rupture and hemorrhage [1–7]. These studies were case reports, which indicated that symptomatic hepatic cysts are still rare.

Different surgical treatments have been proposed for symptomatic patients with hepatic cysts, including percutaneous aspiration, fenestration, hepatic resection, and liver transplantation [8, 9]. Simple large hepatic cysts can be successfully treated by laparoscopic deroofing and alcohol sclerotherapy, but large hepatic cysts that require drainage should be removed surgically to avoid possible infection [3].

In this paper, we describe a case of indolent obstructive jaundice with a 5.0 × 5.3 cm hepatic cyst. The left hepatic duct and common bile duct were blocked by a large pedunculated cystic lump, which was composed of the hepatic cyst wall. The protruded capsular lump was completely cut from the root by using endoscopic needle-knife electrotomy and the patient recovered well without recurrence during the 8-month follow-up period. We also reviewed the literature and discuss the current diagnostic and treatment modalities of hepatic cysts.

## Case presentation

A 41-year-old woman presented to our hospital for further evaluation and management of persistent liver dysfunction, painless xanthochromia, and skin itching for 3 months.

**Fig. 1:**
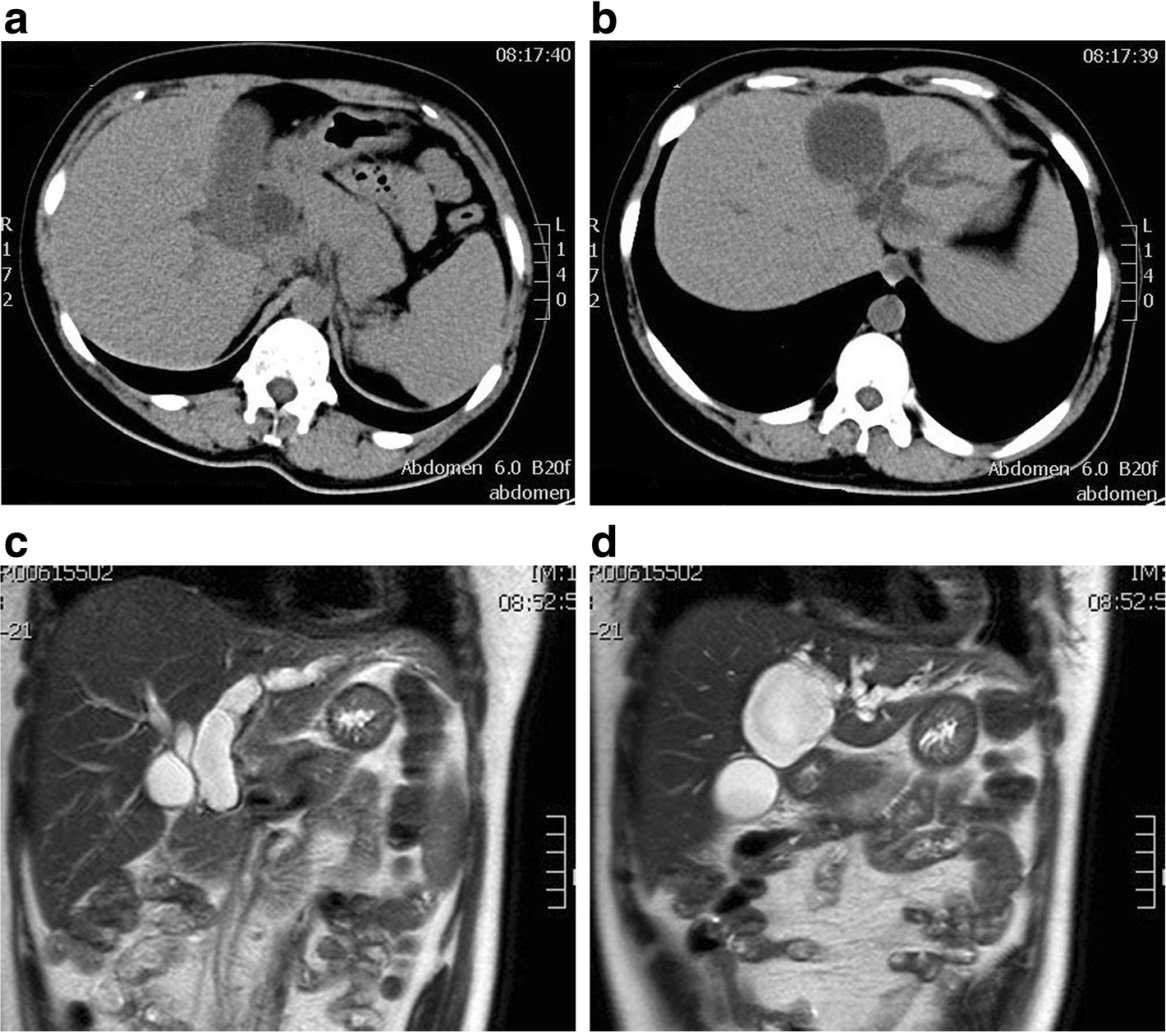
Imaging examination on admission: **a** Epigastric CT shows greatly ectatic common bile duct. **b** Epigastric CT shows greatly left hepatic duct. **c** MRCP shows slightly ectatic right hepatic duct, greatly ectatic left hepatic duct and common bile duct. **d** MRCP shows hepatic hilar region and enlarged gallbladder

On admission, a physical examination showed severe icteric sclera and skin without abdominal tenderness or positive shifting dullness. Liver function showed severe hepatic injury and obstructive jaundice (Table 1). US showed a 5.0 × 5.3 cm, well-circumscribed anechoic lesion with many separations, increased through-transmission of sound, and no evidence of mural nodularity. Epigastric enhanced CT showed a cystic mass with sharply defined margins and smooth, thin, non-reinforced walls in the left medial liver lobe, and obstruction of the biliary tract. The contents of the mass were homogeneous, with water-density lesions, suggesting that it was a cyst. MRI showed a homogeneously hypointense lesion on T1-weighted imaging and this was homogeneously hyperintense on T2-weighted imaging. US, CT, and magnetic resonance cholangiopancreatography (MRCP) showed a slightly ectatic right hepatic duct, a greatly ectatic left hepatic duct and common bile duct, an enlarged gallbladder, and a normal distal end of the choledochus (Fig. 1). Possible reasons for biliary obstruction were bile duct tumor, common bile duct stones, or compression of the common bile duct by a hepatic cyst.

**Table 1:** Changes in liver function during the perioperative period

	ALB(g/L)	TBil(μmol/L)	BC(μmol/L)	ALT(U/L)	AST(U/L)	ALP(U/L)	GGT(U/L)
Preoperative	41.7	407.9	299.5	172	124	560	550
*Post-ERCP*	38.5	76.4	3.2	73	77	157	63
*PST-o*pen operation	46.3	31.7	0.0	33	37	93	36

**Fig. 2:**
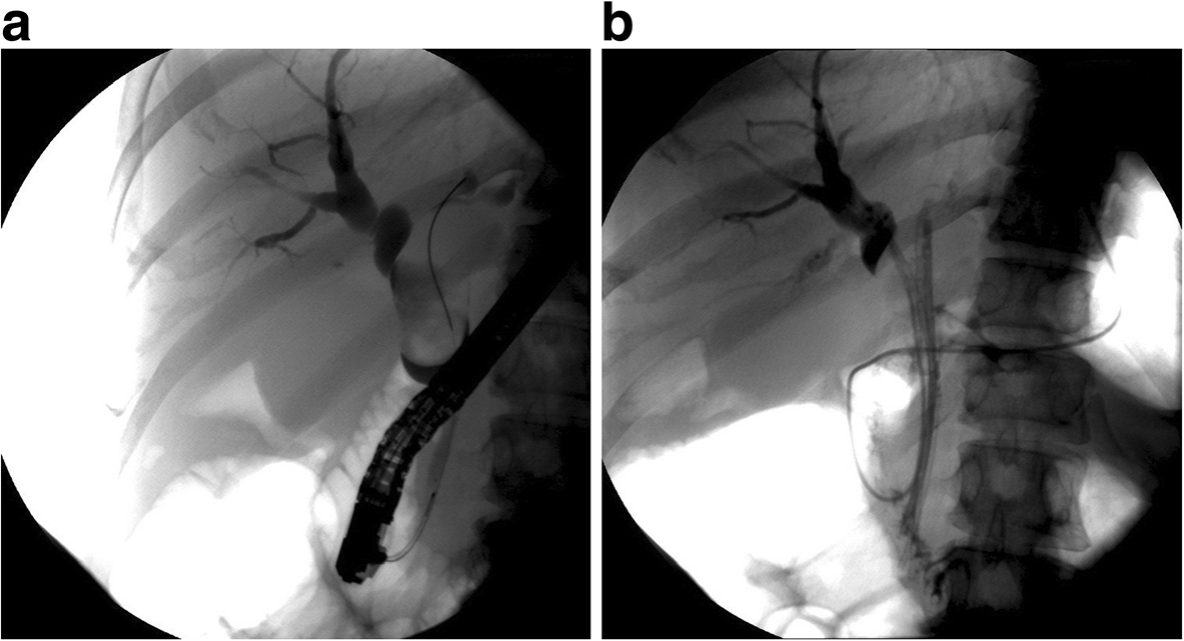
Imaging of endoscopic retrograde cholangiopancreatography: **a** ERC shows that the right hepatic duct is ectatic, but the left hepatic duct and part of the common bile duct cannot be seen. There were no stone, but a lump in the common bile duct was observed, which suggested suspicious cholangiocarcinoma. **b** Two endoscopic retrograde biliary drainages and an endoscopic nasobiliary draingage were placed into the left and right hepatic ducts, respectively, to drain bile

Endoscopic retrograde cholangiography (ERC) was performed to define the cause of obstruction. This procedure showed that the right hepatic duct was ectatic, but the left hepatic duct and common bile duct were not observed. There were no stone, but a large lump was observed in the common bile duct, which suggested suspicious bile duct tumor. Endoscopic sphincterotomy, endoscopic retrograde biliary drainage(ERBD), and endoscopic nasobiliary draingage(ENBD) were successfully performed to drain bile for severe hepatic injury and jaundice, rather than laparotomy, because of coagulation disorders (Fig. 2). After these procedures, the icteric sclera and skin gradually faded, liver function was obviously improved, and coagulation disorders were close to normal levels (Table 1).

**Fig. 3:**
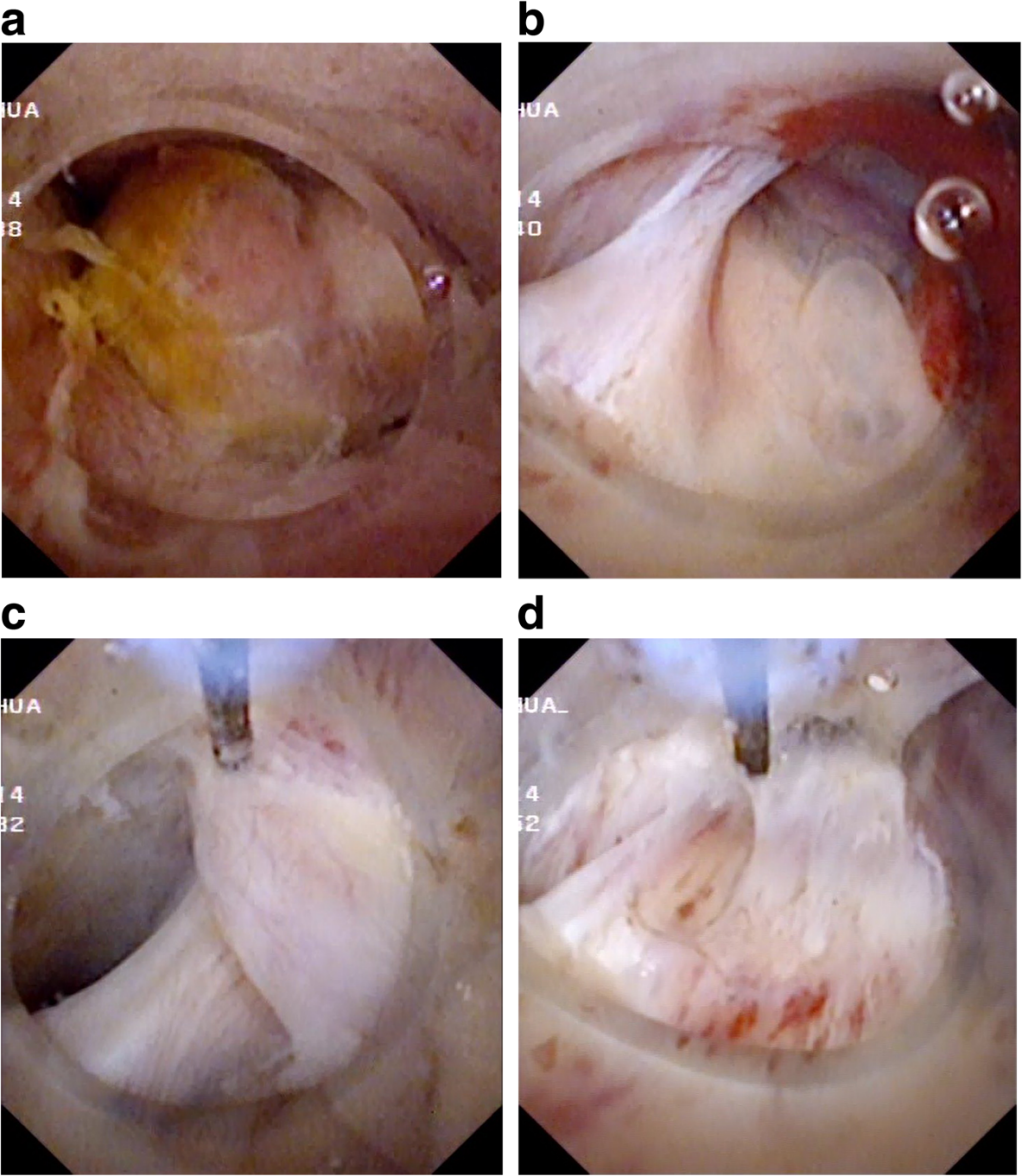
Choledochoscope examination and treatment: **a** The bottom of the lump in the ductuli hepaticus communis. **b** The root of the lump in the left hepatic duct. **c** and **d** Choledochoscopic high-frequency needle-knife electrotomy was performed to remove the lump from the root

**Fig. 4:**
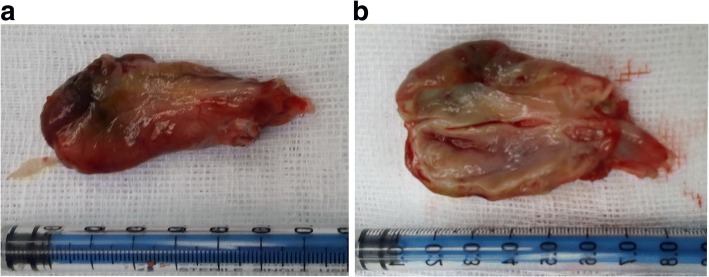
Morphology of the lump: **a** The appearance of the lump was pear shaped with a size of 4 × 6 cm. **b** There was a potential lacuna inside of the lump and the inner wall was smooth, which was similar to the gallbladder, with a capsule wall and capsular space

**Fig. 5:**
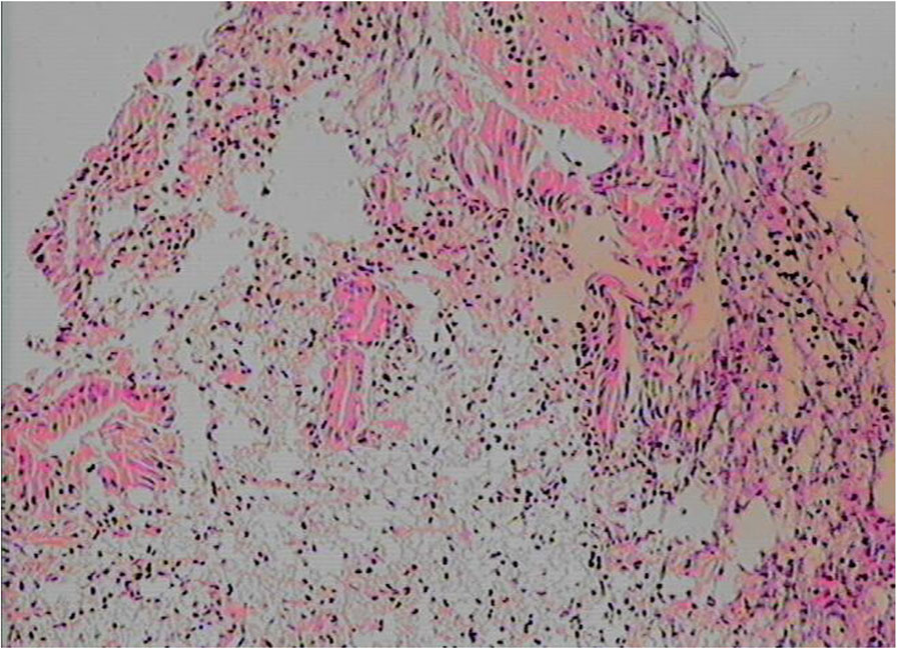
Pathological examination showed that the resected tissue was cystic, and the mucosal structure and inflammatory cell infiltration were observed under microscope. There was no heterocyst or tumor cell (HE X100)

Four weeks later, an open operation was performed for suspicion of bile duct tumor. In exploration of the common bile duct by a choledochoscope, a large pedunculated lump protruding into the common bile duct from the left hepatic duct was discovered. This lump was completely removed from the root of the lump by endoscopic needle-knife electrotomy without active hemorrhage (Fig. 3). The morphology of the lump was similar to the gallbladder with a capsule wall and capsular space (Fig. 4). The choledochoscope was inserted into the hepatic duct from the stump of the lump and there was no viscous liquid secreted by cystadenoma or cystadenocarcinoma. Postoperative pathology of the lump suggested a hepatic cyst wall without heterocysts or tumor cells (Fig. 5). A T tube was retained in the common bile duct in order to prevent bile leakage and observe the recovery of the root incision of the lump and postoperative bleeding.

Two months later, all of the liver function indices recovered to the normal range (Table 1). Epigastric US and CT showed a normal hepatic duct and the hepatic cyst was the same size compared with preoperatively. A choledochoscopic examination was performed through a T-tube fistula. There was no evidence of bile duct tumor. The choledochoscope could enter into the cyst from the defect of the bile duct wall, and the mucosa around the incision recovered well, with no signs of stricture.

**Fig. 6:**
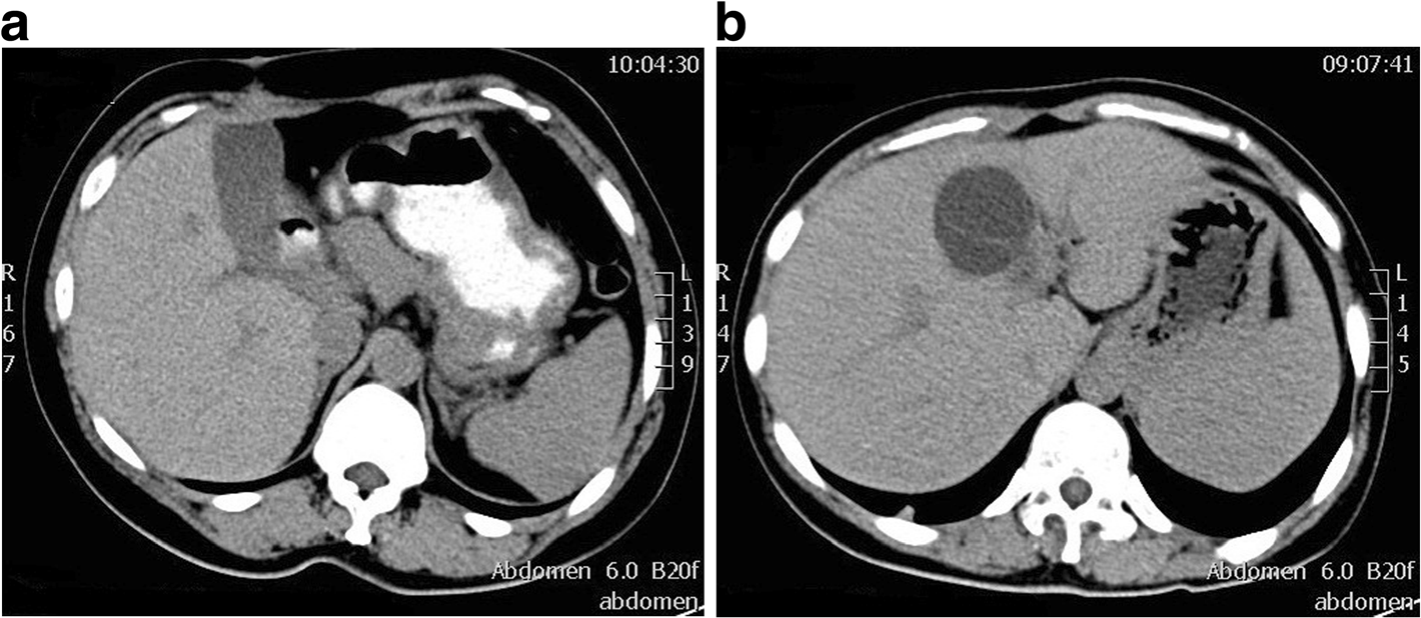
Imaging of epigastric computed tomography 1 year after the open operation: **a** and **b** show a normal hepatic duct and a similar-sized hepatic cyst compared with preoperatively

Three years after the procedure, the patient was in a good general condition, without signs of cholestasis or bile duct stones. The cyst had barely changed (Fig. 6).

## Discussion

On US, CT, and MRI, simple hepatic cysts have the characteristic imaging features of solitary cystic mass. Hepatic cystadenomas and cystadenocarcinomas are more typically multi-locular cystic lesions. The presence of mural or septal nodules, discrete soft-tissue masses, and possibly thick and coarse calcifications increase the likelihood of a cystadenocarcinoma [10–13]. Simple hepatic cysts can also be easily misdiagnosed with other hepatic cystic lesions [14]. In this case, the imaging examinations showed a solitary cystic mass with the features of simiple hepatic cysts. However, the features of many separations slightly, ectatic right hepatic duct, a greatly ectatic left hepatic duct and common bile duct, and an enlarged gallbladder indicated the biliary compression sign of cystadenomas or cystadenocarcinomas.

When simple hepatic cyst is less than 10 cm in diameter, most cystic mass will not cause pressure symptoms in the surrounding organs. However, when jaundice due to biliary obstruction and intermittent upper abdominal pain due to small hepatic cysts are observed, biliary cystadenoma and cystadenocarcinoma should be the primary diagnostic consideration. This is because multi-cystic lesions can invade the hepatic duct and protrude the common bile duct [15–17]. In this case, the patient had presented with persistent liver dysfunction, painless xanthochromia, and skin itching for 3 months. For patients with obstructive jaundice, both percutaneous transhepatic cholangio drainage (PTCD) and ERBD can drain bile and then relieve jaundice and liver injury [18]. The major complications of PTCD were pneumothora, hemorrhag and acidosis caused by persistent loss of bile. Compared with PTCD, ERCP can not only carry out bile internal drainage, but also remove choledocholithiasis. Small sphincterotomy, ENBD and pancreatic stent implantation can reduce the incidence of post-ERCP postoperative bleeding, acute cholangitis and pancreatitis in ERCP [19, 20]. Therefore, ERCP was performed for the patients. ERC showed that the right hepatic duct was ectatic, but the left hepatic duct and common bile duct were not observed and a large pedunculated lump protruding into the common bile duct from the left hepatic duct, which was initially misdiagnosed as bile duct tumor, cystadenomas or cystadenocarcinomas.

Histopathology is still the gold standard for the diagnosis of disease and the extent of bile duct stricture can be observed by choledochoscopy, therefore, an open operation and choledochoscopic examination should be performed. In this case, because of the presence of a common bile duct lump, even though percutaneous aspiration and fenestration could drain the capsule, these treatments could not remove the obstruction. Therefore, left hemihepatectomy was the best option. However, this radical cure was not accepted by the patient’s family for the risk of haemorrhage, leakage and other severe complications [21]. Therefore, we had to use the technology of choledochoscopic high-frequency needle-knife electrotomy to remove the pedunculated lump. This is a new technology that was developed from endoscopic sphincterotomy. We have previously reported this new procedure as a potential complementary medical approach for treating intrahepatic biliary stricture associated with hepatolithiasis and anastomotic strictures after Roux-en-Y hepaticojejunostomy [22, 23]. Choledochoscopic high-frequency needle-knife electrotomy has the effect of cutting and solidification, which can achieve the purpose of separation of tissue and hemostasis by the touch of needle-knife and tissue. Although this technology is proved to be safe, hemobilia is still the most serious complication if the cutting speed is too fast or too deep to destroy the vascular vascular plexus of bile duct wall or parenchyma of the liver.

Fortunately, the lump has a pedicle and the pedunculated lump was completely removed from the root of the lump without hemorrhage, but the capsular space was interlinked to the bile duct and bile could flow into the capsule. This is similar to cysts of the bile duct, but the results were good. Follow up at 3 years showed that the patient was in a good general condition, without signs of cholestasis or bile duct stones. The cyst had barely changed at this time.

## Conclusion

Hepatic cyst wall protruding into the common bile duct can form capsular lump and result in indolent jaundice. Choledochoscopic high-frequency needle-knife electrotomy could be considered as a simple, safe and effective complementary approach for benign mass on the bile duct wall.

## Abbreviations

CT: Computed tomography
ERC: Endoscopic retrograde cholangiography
MRCP: magnetic resonance cholangiopancreatography
MRI: Magnetic resonance imaging
US: Ultrasonography
